# Supplementary material for: Multivalent polymers can control phase boundary, dynamics, and organization of liquid-liquid phase separation
Source: PLoS One. 2021 Nov 8;16(11):e0245405. doi: 10.1371/journal.pone.0245405 (PMC8575181; doi:10.1371/journal.pone.0245405)
Supplement: S1 File — This file contains simulation results of the monovalent binding affinity, an example system energy profile over time, and details of our neighbor persistence calculations. (PDF) [file pone.0245405.s001.pdf]

Supporting Information: Multivalent polymers can control phase boundary, dynamics, and organization of liquid-liquid phase separation

E. Zumbro and A. Alexander-Katz

1 Monovalent binding affinity

To confirm that we used biologically relevant binding affinities for individual binding site interactions, we placed increasing concentrations of monovalent monomers (free monomeric beads of the same type as make up the polymer) in a box with a single monovalent target. At each concentration of monomer beads, we measured the fraction of time the target spent bound ϕ. The plot of the fraction of time the target spent bound versus the monomer bead concentration is shown in Figure S1. To estimate the K_D of binding, we fit our simulation data with the Langmuir adsorption curve $\phi = \frac{[P]}{[P]+K_\text{D}}$ where $[P]$ is the concentration of monomeric polymer beads and K_D is the dissociation constant of the binding reaction. We converted our unitless K_D to a concentration in Molar by estimating our target diameter to be approximately 5 nm [1].

This method resulted in a $K_\text{D} = 1\times10^{-4}$ M for $\Delta E_0 = -4k_\text{B}T$. This is within the typical binding site affinity range for protein-protein and protein-RNA binding found in biocondensates [2–4].

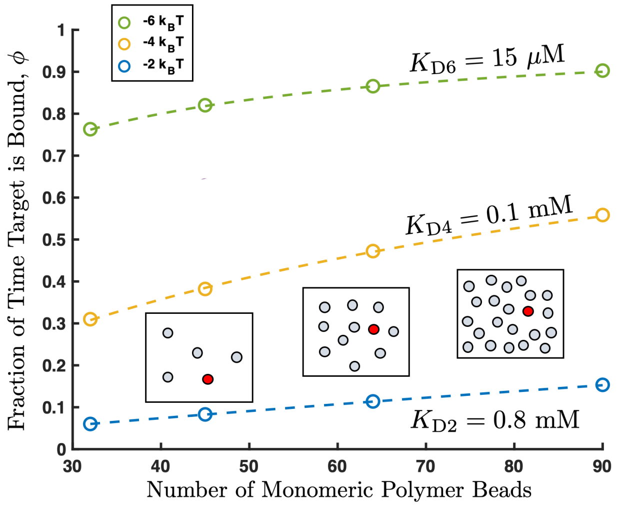

Figure S1: Fraction of time a monovalent target spent bound to monomers versus the number of available binding monomers. In the schematic, the target is shown in red and the monomers are shown in light grey-blue. Simulation values are shown as circles (o) with the dashed line (- - -) showing the fit of the Langmuir adsorption curve for $K_\text{D} = 0.1$ mM. This figure is adapted from Zumbro *et al.* with permission [5].

2 System energy profile

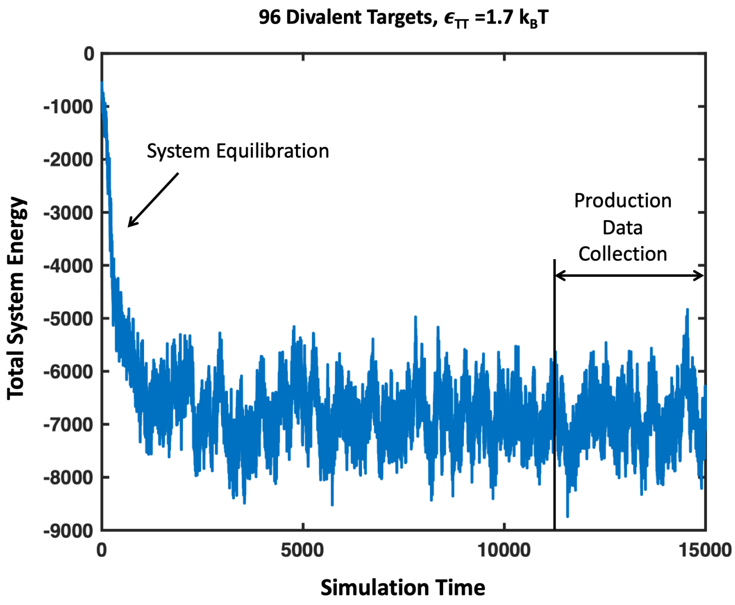

Figure S2: Example of a typical system energy profile over time. The total energy is shown for a system with four 16mer polymers and 96 divalent targets with a target-target attraction $\epsilon_{\mathrm{TT}} = 1.7 k_{\mathrm{B}}T$. Simulations are run for 1.5×10^8 time steps with simulation energy shown above for every 10^4 time steps where each time step $\Delta\tilde{t} = 10^{-4}$ characteristic diffusion time. There is an initial large drop in energy while the system equilibrates. Production research data is taken from the last quarter of the simulation, past this equilibration time period. This figure is adapted from Zumbro *et al.* with permission from Elsevier [6].

3 Neighbor Persistence

We consider a target protein bead to have a neighbor if the distance between the bead and any other target bead $r_{ij} \leq 2.5a$ where a is the radius of a target bead, and $r_{ij} = |\mathbf{r}_i - \mathbf{r}_j|$ is the distance between the centers of beads i and j [7]. Therefore the matrix of nearest neighbors $\mathcal{M}$ is:

$$\mathcal{M}(i,j) = \begin{cases} 1 & \text{if} \quad \mathrm{r_{ij}} \leq 2.5a \\ 0 & \text{otherwise} \end{cases} \tag{1}$$

At each time step, the neighbor persistence (np) is the fraction of original neighbors that remain neighbors over that time interval τ:

$$np = \frac{\sum_{i,j}^{N_{\mathrm{T}}} \mathcal{M}_{t_0}\mathcal{M}_{t_0+\tau}}{\sum_{i,j}^{N_{\mathrm{T}}} \mathcal{M}_{t_0}} \tag{2}$$

where N_{T} is the total number of target proteins in the simulation, and t_0 is the start of the time interval. This measures how quickly target beads exchange their neighbors. This number should decay to zero quickly (at short τs) if droplets do not form or if the droplets are more liquid-like. np should decay slowly or never reach zero if the droplet is more solid-like, meaning that the bead has the same neighbors over a long time period τ.

References

[1] Veronica H Ryan, Gregory L Dignon, Gül H. Zerze, Charlene V. Chabata, Rute Silva, Alexander E. Conicella, Joshua Amaya, Kathleen A Burke, Jeetain Mittal, and Nicolas L. Fawzi. Mechanistic View of hnRNPA2 Low-Complexity Domain Structure, Interactions, and Phase Separation Altered by Mutation and Arginine Methylation. *Molecular Cell*, 69(3):465–479.e7, feb 2018.

[2] Pilong Li, Sudeep Banjade, Hui-Chun Cheng, Soyeon Kim, Baoyu Chen, Liang Guo, Marc Llaguno, Javoris V. Hollingsworth, David S. King, Salman F. Banani, Paul S. Russo, Qiu-Xing Jiang, B. Tracy Nixon, and Michael K. Rosen. Phase transitions in the assembly of multivalent signalling proteins. *Nature*, 483(7389):336–340, mar 2012.

[3] Yousif Shamoo, Norzehan Abdul-Manan, Ann M Patten, Janet K Crawford, Matthew C Pellegrini, and Kenneth R Williams. Both RNA-Binding Domains in Heterogeneous Nuclear Ribonucleoprotein A1 Contribute Toward Single-Stranded-RNA Binding. *Biochemistry*, 33(27):8272–8281, jul 1994.

[4] Yousif Shamoo, Norzehan Abdul-Manan, and Kenneth R Williams. Multiple RNA binding domains (RBDs) just don't add up. *Nucleic Acids Research*, 23(5):725–728, 1995.

[5] Emiko Zumbro and Alfredo Alexander-Katz. Influence of Binding Site Affinity Patterns on Binding of Multivalent Polymers. *ACS Omega*, 5(19):10774–10781, may 2020.

[6] Emiko Zumbro and Alfredo Alexander-Katz. Polymer Stiffness Regulates Multivalent Binding and Liquid-Liquid Phase Separation. *Biophysical Journal*, oct 2020.

[7] Alfredo Alexander-Katz, Hirofumi Wada, and Roland R Netz. Internal Friction and Nonequilibrium Unfolding of Polymeric Globules. *Physical Review Letters*, 103(2):028102, jul 2009.
